# Obituary

**Published:** 2010

**Authors:** NK Pradhan, HKT Raza

**Affiliations:** IMA House, Ranihat Medical Road, Cuttack – 753 007, Orissa, India; 1 Regional Spinal Injuries and Rehabilitation Centre, Medical College Campus, Jabalpur, India. E-mail: nkp@ioacon2009.net, hktraza@yahoo.com

**Figure:**
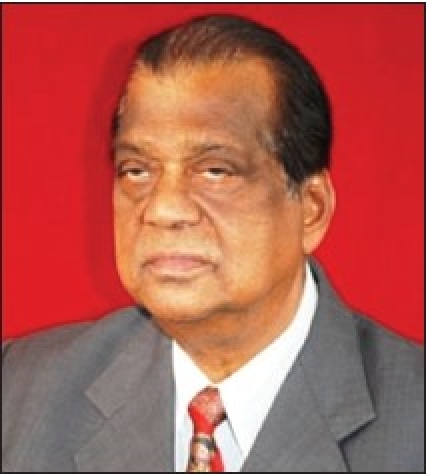
Dr. P. Tejeswar Rao (03.09.1934 - 31.01.2010)

The measure of a well-lived life is not material gain but the lives touched upon and the sorrow left behind. If the outpour of emotions on his loss is any indication, and it most certainly is, we can proudly and confidently say that Dr. P. Tejeswar Rao's life was indeed well-lived. Dr. P. Tejeswar Rao passed away in the early hours (4 am) of 31^st^ January 2010.

Dr. P. Tejeswar Rao, MBBS (Hons), FRCS (London), FRCS (Edinburgh), M.CH (L'Pool) was born on 3^rd^ September 1934 in Aska, Ganjam district, Orissa. He had a brilliant academic career at Khalikote College, Berhampur. In 1958, he graduated from SCB Medical College, Cuttack, with a distinguished undergraduate career, Honours in three subjects and four Gold medals in final MBBS; he was crowned the Best Graduate of the year. He obtained FRCS (London), FRCS (Edinburgh) in general surgery but later developed interest in M.Ch in Orthopedics. He returned to Orissa in 1964 to begin his surgical career at SCB Medical College Hospital, Cuttack. He joined as Lecturer in Orthopedics in 1965 and went on to become Professor and Head of Orthopedics in 1974. With enthusiasm and zeal he built the three-storied separate Orthopedic Department even before Government approved it. In 1975, he was transferred to MKCG Medical College Berhampur. He returned to Cuttack in 1985 as Professor and Head of Orthopedics, to become Superintendent, SCB Medical College and Hospital and Director of Medical Education and Training, Orissa, before retiring on 30^th^ September 1992.

Divinely blessed with plenty of wealth and knowledge, a simple man with a towering personality and amicable nature, Dr. Rao was liked by all and left no stone unturned in bringing Orthopedics to new heights in the State. A great surgeon and academician, he was famous for his punctuality and involvement in all academic forums besides his busy schedule from dawn to dusk with his professional activity till his last breath.

He was a life member of several academic forums of National and International societies like Association of Surgeons of India (ASI), Indian Orthopaedic Association (IOA), World Orthopedic Concern (WOC), Société Internationale de Chirurgie Orthopédique et de Traumatologie (SICOT); he was president of Indian Medical Association (IMA), state branch, twice. He earned name and fame participating in scientific deliberations, debates and discussions, sharing his professional experience at all academic meets of IMA, ASI and encouraging juniors for greater cause of mankind.

He delivered the prestigious Silver Jubilee Oration in IOACON 1990 and Kini Memorial Oration in 1992. He was the President of the IOA in 1991. His love for Indian Orthopaedic Association and its activities was well known. He was keen to start an academic activity during the IOACON's. In the IOACON 2003 at Chennai the “P. Tejeswar Rao Gold Medal” for the best paper by a Post Graduate was started with a handsome donation from him. He made it a point of attending the inaugural as well as every post graduate competition subsequently – often acting as a judge to encourage the post graduates.

He was instrumental in forming the Orissa Orthopedic Association (OOA) in 1976 and nurturing it to have its present strength and its own office building at Cuttack. His personality and popularity allured more meritorious students to the PG in orthopedics. He brought OOA to national fame by organizing IOACON 1986 at Cuttack as the organizing secretary and, in 2009, to Bhubaneswar as organizing chairman, as if it was ordained on him to do – and it was done so well.

Dr. P. Tejeswar Rao is survived by his wife, P. Vijayalaxmi, two sons, a daughter and grand children. We pray to the Almighty let his soul rest in peace.

